# Ribavirin is effective against drug-resistant H7N9 influenza virus infections

**DOI:** 10.1007/s13238-016-0287-0

**Published:** 2016-07-18

**Authors:** Yuhai Bi, Gary Wong, Yingxia Liu, Lei Liu, George F. Gao, Yi Shi

**Affiliations:** 1 CAS Key Laboratory of Pathogenic Microbiology and Immunology, Institute of Microbiology, Chinese Academy of Sciences, Beijing, 100101 China; 2 Shenzhen Key Laboratory of Pathogen and Immunity, State Key Discipline of Infectious Disease, Shenzhen Third People’s Hospital, Shenzhen, 518112 China; 3 Center for Influenza Research and Early-warning (CASCIRE), Chinese Academy of Sciences, Beijing, 100101 China; 4 Research Network of Immunity and Health (RNIH), Beijing Institutes of Life Science, Chinese Academy of Sciences, Beijing, 100101 China; 5 Collaborative Innovation Center for Diagnosis and Treatment of Infectious Disease, Zhejiang University, Hangzhou, 310003 China

**Dear Editor,**

In February and March 2013, a novel influenza A (H7N9) virus emerged in China, causing an acute respiratory distress syndrome and occasionally multiple organ failure with high fatality rates in humans (Li et al., 2014). A total of 681 laboratory-confirmed cases and 275 deaths have been reported as of November 13th, 2015, with a fatality rate of 40% (http://www.who.int/influenza/human_animal_interface/HAI_Risk_Assessment/en/). H7N9 has been evolving and established amongst chickens in China over the past two years with occasional human infections (Lam et al., 2015; Su et al., 2015), thus posing a threat to public health. In the absence of an annually-updated effective vaccine, antiviral drugs constitute the first line of defense against H7N9 infections. H7N9 viruses already possess natural resistance to M2-ion channel blockers (amantadine and rimantadine) when it first emerged in 2013 (Gao et al., 2013). Therefore neuraminidase inhibitors (NAIs), which include oseltamivir (TamifluH), zanamivir (RelenzaH) and peramivir constitute the main antiviral drugs against H7N9 infections (Hu et al., 2013; Wu et al., 2013). However, treatment with NAIs against H7N9 infections has resulted in the emergence of drug-resistant mutant viruses, as soon as 1~9 days after administration (Gao et al., 2013; Hu et al., 2013). Moreover, the first H7N9 isolate (A/Shanghai/1/2013(H7N9), SH-H7N9) was resistant to oseltamivir (Gao et al., 2013).

It is therefore necessary to investigate whether other classes of drugs can control H7N9 infections. A recent study shows that the RNA polymerase 2 (PB2) gene of the H7N9 virus is critical for virulence in mammals (Bi et al., 2015). Ribavirin is a well-characterized, broad-spectrum nucleoside inhibitor used to halt the synthesis and capping of viral RNA and mRNA, respectively, by the viral RNA-dependent RNA polymerase (Crotty et al., 2000). Ribavirin is approved for treating infections with Hepatitis C virus and respiratory syncytial virus (Graci and Cameron, 2006). Moreover, ribavirin is effective by itself or in combination with NAIs and/or M2-ion channel blockers against H1N1, H3N2 and H5N1 influenza infections (Smee et al., 2005; Ilyushina et al., 2008; Nguyen et al., 2009). Here, we want to investigate whether ribavirin is effective by itself against H7N9 virus infections, especially to virus mutants that have developed resistance to NAIs. To compare the efficacy of ribavirin to that of NAIs, zanamivir was used as a positive control.

To determine the effectiveness of the ribavirin against H7N9 viruses *in vitro*, NAI-sensitive (A/Anhui/1/2013, AH-H7N9) and -resistant (SH-H7N9) viruses were used in this study. The 50% effective virus-inhibitory concentration (EC_50_) value was used to evaluate the antiviral functions of ribavirin. Serial two-fold dilutions of ribavirin were used to test the EC_50_ against AH-H7N9 and SH-H7N9 on Madin-Darby Canine Kidney (MDCK) epithelial cells, respectively. The results showed that ribavirin was effective against both AH-H7N9 and SH-H7N9 with an EC_50_ of 0.01~0.02 mg/mL (3~4 nmol) (Table 1), implying that ribavirin could be utilized against both NAI-sensitive and -resistant H7N9 viruses. In addition, the ribavirin at a dose of ~300 folds of the calculated EC_50_ is also safe *in vitro* without significant cytotoxicity.

**Table 1:** EC_50_ values of ribavirin against H7N9 virus

Viruses	Ribavirin (±SD)	
	mg/mL	Effective dose (nmol)
AH-H7N9	0.0166 (±0.0029)	3.3964 (*±*0.5864)
SH-H7N9	0.0182 (±0.0021)	3.7186 (*±*0.4288)

*In vitro* studies showed that the ribavirin is effective to both the NAI-sensitive (AH-H7N9) and -resistant (SH-H7N9) viruses. To further study antiviral function *in vivo*, the efficacy of ribavirin against H7N9 infection was then tested in a mouse animal model. To confirm the antiviral functions of ribavirin, zanamivir was selected as a positive drug control.

The survival, percentage weight change and clinical symptoms in the animals after challenge were monitored over the course of the experiment (14 days). All animals infected with AH-H7N9 displayed ruffled fur, loss of activity and body weight loss beginning 2 days post-infection (d.p.i.). Mice treated with placebo did not survive and had weight loss of over 35% (Fig. 1A and 1B). Mice treated with ribavirin or zanamivir also experienced weight loss of up to around 30%, but all mice in each group gradually recovered and survived (Fig. 1A and 1B). In general, the clinical signs of mice in the ribavirin and zanamivir groups were milder than that of the placebo group. No significant changes were observed from mice in the mock-infection group.

**Figure 1:**
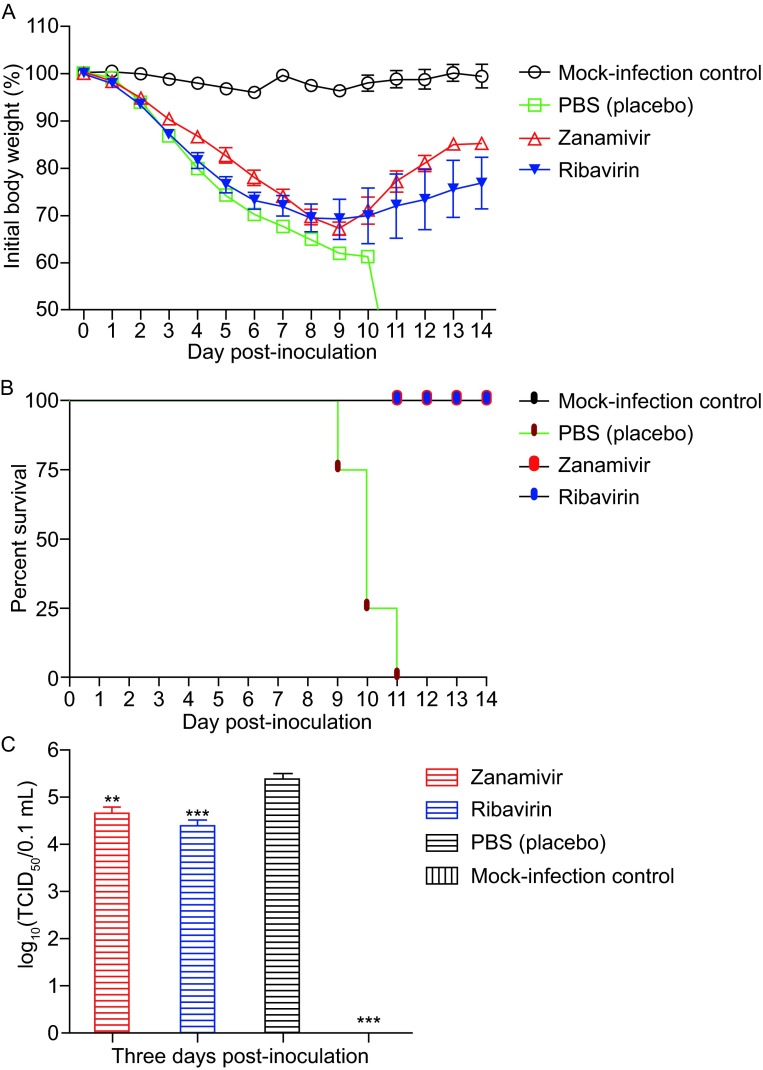
Efficacy of ribavirin in mice infected with AH-H7N9. Mice (*n* = 9) were given ribavirin, zanamivir or PBS (placebo) twice daily, respectively. Animals were inoculated i.n. with 10× LD_50_ of AH-H7N9 in 50 µL PBS at one day after the initiation of treatment, and an untreated group was mock-challenged with an equal volume of PBS as control. Survival and body weights were monitored daily over a 14-day observation period and expressed as percentages of the initial values (A). The mortality rate was calculated from the survival curve of each group (B). Five mice from each group were euthanized at 3 d.p.i. LVTs were quantified in MDCK cells, and expressed as log_10_ TCID_50_/0.1 mL (C). The data are presented as the mean ± SD. Statistical analysis on the LVTs were performed with a paired-sample *t*-test (*, *P* < 0.05; **, *P* < 0.01; ***, *P* < 0.001)

Five mice from each group were euthanized at 3 d.p.i. and the lung virus titers (LVTs) were test in MDCK cells, with the results presented as 50% tissue culture infective dose (TCID_50_). The results showed that the LVTs from mice treated with either the ribavirin or zanamivir were 10-fold lower than that of the sterile phosphate-buffered saline (PBS) placebo group (*P* < 0.5) (Fig. 1C). Interestingly, ribavirin-treated mice displayed lower LVTs than that of zanamivir-treated animals, but the values were not statistically significant (*P* > 0.5) (Fig. 1C).

In conclusion, ribavirin displayed antiviral activities comparable to zanamivir against H7N9 virus infections *in vitro* and *in vivo*, and the data support the use of ribavirin against NAI-resistant H7N9 virus infections. Since it had been shown in previous studies that SH-H7N9 containing the NAI-resistant K224 mutation (H3 numbering) on the neuraminidase (NA) gene reverts back to the NAI-sensitive R224 mutation *in vivo* (Yen et al., 2014), as well as the relatively lower replication ability *in vitro* compared to AH-H7N9 (the NAI-sensitive virus) (Wu et al., 2013), only AH-H7N9 was used for the studies in mice.

Notably, administration of ribavirin via the intranasal (i.n.) route was still efficacious against severe influenza virus infections without any of the negative side effects associated with oral or intravenous administration (Gilbert and McLeay, 2008). The present study showed that the i.n. route is also effective in the case of ribavirin against H7N9 virus infection. Human infections with H7N9 mainly originate from contact with infected poultry or contaminated materials in live poultry markets (Gao, 2014), and administration of ribavirin into the nasal mucosa would be an effective strategy to mitigate the risk of H7N9 infections in humans.

## FOOTNOTES

This work was supported by the National Basic Research Program (973 Program) (Nos. 2013CB531502 and 2014CB542503) and the National Natural Science Foundation of China (Grant No. 31402196). Yi Shi is supported by the Excellent Young Scientist Program of the Chinese Academy of Sciences and the Youth Innovation Promotion Association CAS (2015078). Gary Wong is the recipient of a Banting Postdoctoral Fellowship from the Canadian Institutes of Health Research (CIHR) and the President’s International Fellowship Initiative from the Chinese Academy of Sciences (CAS).

Ribavirin (National Drug Approval No. H20043189) was obtained from Penglai Nuokang Pharmaceutical Co., Ltd. (Penglai city, Shandong Province, China). Zanamivir carboxylate was purchased from Shandong Xiya Chemical Industry Co., Ltd. (Shandong Province, China).

Yuhai Bi, Gary Wong, Yingxia Liu, Lei Liu, George F Gao and Yi Shi declare that they have no conflict of interest. All institutional and national guidelines for the care and use of laboratory animals were followed.

## Electronic supplementary material

Below is the link to the electronic supplementary material.

Supplementary material 1 (PDF 92 kb)
