# Supplementary material for: Ribavirin is effective against drug-resistant H7N9 influenza virus infections
Source: Protein Cell. 2016 Jul 18;7(8):611–4. doi: 10.1007/s13238-016-0287-0 (PMC4980329; doi:10.1007/s13238-016-0287-0)
Supplement: Supplementary file 1 — Supplementary material 1 (PDF 92 kb) [file 13238_2016_287_MOESM1_ESM.pdf]

Supplemental Materials

Materials and methods

Compounds

Ribavirin (National Drug Approval No. H20043189) was obtained from Penglai Nuokang Pharmaceutical Co., Ltd. (Penglai city, Shandong Province, China). Zanamivir carboxylate was purchased from Shandong Xiya Chemical Industry Co., Ltd. (Shandong Province, China).

Viruses

A/Anhui/1/2013(H7N9) and A/Shanghai/1/2013(H7N9) viruses were generated by reverse genetics as described previously (Bi et al., 2015; Wu et al., 2013). Briefly, all eight gene segments from A/Anhui/1/2013 (H7N9) and A/Shanghai/1/2013(H7N9) were cloned into a dual-promoter plasmid. 293T and MDCK cells mixed in a ratio of 5:1 were transfected with eight genetic plasmids of each virus. At 48 hours post-transfection, the cell supernatant was inoculated in 9-10-day-old specific pathogen free (SPF) chicken embryos to produce stock viruses. Each viral segment was amplified by reverse transcription-PCR (RT-PCR) and sequenced to confirm the identity of the virus.

In vitro studies

The antiviral activity of ribavirin was determined in MDCK cells. The 50% effective (virus-inhibitory) concentration (EC_{50}) was calculated by Reed and Muench method (Reed and Muench, 1938) based on the inhibition of virus-induced cytopathic effects (CPEs) coupled with hemagglutinination test. Two-fold serial dilutions of ribavirin or zanamivir from 10 mg/ml to 0.000305 mg/ml were evaluated against each virus in 96-well microplates, with approximately 10^4 MDCK cells/well. Two hundred times the 50 % $TCID_{50}$ of virus (200 x $TCID_{50}$) was mixed with an equal volume of ribavirin dilution at 37 ℃ for 1 h, and then were added onto cells. CPEs and the HA titers were detected after incubation at 37 ℃ for 72 h for EC_{50} values.

In vivo studies

Seven-week old, female BALB/c mice (Vital River Laboratory, Beijing, China) (n=36) were randomly divided into four groups of nine animals each. Mice were anesthetized with inhalational CO_2 and were then given either ribavirin or zanamivir (20 mg/kg) dissolved in 50 μL of PBS twice daily via the i.n. route for 9 days, or an equal volume of sterile PBS as a placebo treatment. One day after the initiation of treatment, the mice were challenged i.n. with 50 μL of 10 times the median lethal dose (10 x LD_{50}) AH-H7N9 diluted in PBS. A group was given an equal volume of PBS replacing the drug and virus as a mock-infection control. All of the mice were monitored daily for general behavior and clinical signs, including food intake, body weight, inactivity, and mortality for 14 days. Mice that lose $\geq$ 35% of its preinoculation body weight were euthanized in accordance with animal ethics guidelines.

Five mice from each group were euthanized at 3 d.p.i., and their lungs were collected and homogenized using a QIAGEN TissueLyser II machine (30 cycles/second for 4 minutes) in 1 ml of ice-cold PBS under sterile conditions. Solid debris was then pelleted by centrifugation at 5, 000 × g for 10 min, and the supernatants were used for virus titrations in MDCK cells. The $TCID_{50}$ value was calculated by Reed and Muench method (Reed and Muench, 1938), based on the CPE and HA titers after the incubation at 37 ℃ for 72 h.
